# Identification of Ferroptosis-related Genes Associated with the Prognosis of Hepatocellular Carcinoma

**DOI:** 10.1007/s12013-026-02096-y

**Published:** 2026-06-25

**Authors:** Kai Yan, Fangfang Hu, Haodong Tang, Jiahua Zhou

**Affiliations:** 1 Nanjing Medical University, Nanjing, 210009 Jiangsu China; 2 Department of Hepatobiliary and Pancreatic Surgery, Medical School, Zhongda Hospital, Southeast University, No. 87 Dingjiaqiao, Gulou District, Nanjing, 210009 China; 3 Medical School, Southeast University, Nanjing, 210009 China

**Keywords:** Cell cycle, Competitive endogenous RNA, *DDX11*-*AS1*, Ferroptosis, Hepatocellular carcinoma

## Abstract

Ferroptosis has been increasingly implicated in the progression of various tumors, including hepatocellular carcinoma (HCC). This study aimed to identify ferroptosis-related genes with prognostic significance in HCC. Differential expressed genes (DEGs) and long non-coding RNAs (DElncRNAs) were identified from TCGA LIHC RNA-seq data. A competitive endogenous RNA (ceRNA) network was established based on ferroptosis-related lncRNA-mRNA pairs. Prognostic genes were identified through survival analysis, and selected DEGs were further validated via quantitative PCR. Co-expression analysis identified 218 lncRNA-mRNA pairs, including 216 DEGs and 11 DElncRNAs. Among these, 11 upregulated ferroptosis-related genes were identified as predicted targets of the lncRNA DDX11-AS1. MiR-195 and miR-424 both regulated CDC25A and HELLS. Elevated expression of DDX11-AS1, CDC25A, EZH2, FANCD2, and HELLS was associated with poorer survival. In vitro cellular experiments showed that the knockdown of DDX11-AS1, CDC25A, and HELLS inhibited cell proliferation and invasion by promoting ferroptosis in Huh7 cells. The 11 ferroptosis-related genes and DDX11-AS1 may serve as valuable prognostic biomarkers and potential immunotherapeutic targets in HCC.

**Supplementary Information:**

The online version contains supplementary material available at https://doi.org/10.1007/s12013-026-02096-y.

## Introduction

 Hepatocellular carcinoma (HCC) represents the most frequent histological form of primary liver cancer [1]. Its morbidity is associated with various factors, including viral infection, obesity, diabetes, and a medical history of alcohol consumption, smoking, and aflatoxin exposure [2]. The incidence and mortality of HCC are strikingly high, with approximately 660,000 new deaths are reported worldwide annually [3]. Despite liver transplantation being an effective intervention, the tumor recurrence and metastasis rates remain high, leading to poor overall survival. Consequently, it remains imperative to investigate the mechanisms underlying HCC development, progression, and novel therapeutic approaches.

The pathogenesis of HCC involves dysregulation across multiple layers of molecular control, including genes, non-coding RNAs, and epigenetic regulation [4, 5]. For example, Li et al. [6] demonstrated that the long non-coding RNA (lncRNA) *DDX11-AS1* promotes tumor cell growth and invasion in HCC. Similarly, the downregulation of serum *miR-424* in HCC patients is associated with elevated alpha-fetoprotein (AFP) level and tumor cell invasion. Collectively, these factors represent promising prognositic biomarkers or the therapeutic targets for HCC.

Emerging research has established a significant link between ferroptosis and tumors. Ferroptosis is a novel form of regulated cell death that modulates critical processes in human tumors such as tumor immune microenvironment and progression [7, 8]. Notably, the expression profiles of genes related to ferroptosis may serve as predictors for the overall survival in HCC patients [9]. The well-known epigenetic regulator Enhancer of zeste homolog 2 (*EZH2*)gene has also been shown to influence ferroptosis [10]. Recent research shows that *EZH2* regulates ferroptosis-related genes directly or indirectly, thereby affecting this cell death pathway [11, 12]. These findings collectively indicate that focusing on mechanisms related to ferroptosis may provide novel mechanisms for HCC.

This study aimed to characterize the ferroptosis landscape and its prognostic relevance in HCC. Using transcriptomic data, differentially expressed lncRNAs and genes related to ferroptosis were identified and utilized to construct a competitive endogenous RNA (ceRNA) regulatory network. Furthermore, prognostic genes associated with ferroptosis were further identified and a ferroptosis-related signature predictive of the overall survival of HCC would be identified. Collectively, this work positions ferroptosis-related RNAs as promising therapeutic targets in HCC. This study is reported following the REMARK checklist.

## Materials and Methods

### Data and Processing

To profile the transcriptome of HCC, we utilized Bulk RNA and clinical data for the Liver Hepatocellular Carcinoma (LIHC) cohort from The Cancer Genome Atlas (TCGA) database, comprising 374 cancer samples and 50 adjacent normal controls. RNAs with an average expression of less than one were excluded from subsequent analysis. Differentially expressed lncRNAs (DElncRNAs) and mRNAs (DEGs) were screened using the edgeR package [13]. Criteria were set as |log_2_ fold change (FC)| ≥ 2, *p* < 0.05, and false discovery rate (FDR) < 0.05. For miRNA expression analysis, the cutoff criteria were set as |log_2_FC| ≥ 1 and FDR < 0.05. Additionally, an independent validation dataset GSE36376 (GPL10558), containing 193 non-tumor tissues and 240 tumor tissues, was acquired from the Gene Expression Omnibus [14].

### Analysis of lncRNAs and mRNAs with Co-expression Profiles

To identify co*-*expression relationships between DElncRNAs and DEGs in LIHC samples, Pearson correlation analysis was performed, pairs with a correlation coefficient *r* > 0.4 and *p* < 0.05 were identified. Subsequently, the identified DEGs were submitted to functional annotation using the DAVID database [15]. Functional terms were considered significantly enriched if they encompassed at least two genes and met a significance threshold of *p* < 0.05.

### Screening of Genes Related to Ferroptosis

Firstly, ferroptosis-related factors (drivers, suppressors, markers, and unclassified) were obtained from The FerrDb database [11]. The intersection between these genes and the previously identified DEGs was defined as the set of ferroptosis-related DEGs for subsequent analysis.

### Identification of Prognostic lncRNAs, mRNAs, and Clinical Factors

Cox regression analysis was performed to identify DElncRNAs, DEGs, and clinical variables associated with HCC prognosis. A total of 342 samples with complete transcriptome data and clinical survival information were included to evaluate the prognostic relevance of DElncRNAs, DEGs, and clinical factors, such as sex, race, age, and clinical stage. The significance criterion was set at log-rank p-value < 0.05.

### CeRNA Regulatory Network Analysis for Ferroptosis-related DEGs

To elucidate the post-transcriptional regulation of ferroptosis-related DEGs, miRNA-mRNA regulatory interactions were predicted using the starBase database (version 3.0). The final ceRNA regulatory network incorporating lncRNA, miRNA, and mRNA was constructed using Cytoscape 3.8.0 [12].

### Survival Analysis of Prognostic RNAs

Finally, the prognostic significance of previously identified RNAs was evaluated using overall survival analysis on GEPIA 2.0. Tumor samples were stratified into two comparative groups (high- and low-expression) based on the median expression levels. The significant criterion was set at log-rank p-value < 0.05.

### Cells, Culture Conditions and Treatments

Human HCC cell lines (HepG2, LM3 and Huh7) were purchased from Sunncell (China). HepG2 were cultured in HepG2-specific growth medium (Sunncell), while Huh7 (Sunncell) and LM3 cells were maintained in DMEM (Gibco) supplemented with 10% fetal bovine serum (FBS) and 1% penicillin-streptomycin. Human MIHA hepatocytes were cultured in RPMI-1640 medium (Procell) containing 10% FBS and 1% penicillin-streptomycin at 37 °C with 5% CO2. All cells were cultured under the same conditions at 37 °C in an atmosphere of 5% CO2.

### Western Blot Analysis

Total proteins were extracted from cells using RIPA lysis buffer (Beyotime) and quantified using a BCA Protein Assay Kit (Beyotime). Equal amounts of protein separated by 10% SDS-PAGE (Beyotime), and then electrotransferred onto PVDF membranes (constant voltage: 65 V for 2 h). Following blocking with 5% non-fat milk at room temperature for 1 h, the membranes were incubated overnight at 4 °C with the following primary antibodies: anti-CDC25A (1:1000, Abconal, A1173), anti-HELLS (1:1000, CST, USA), anti-GPX4 (1:1000, Affinity), anti-SLC7A11 (1:1000, Affinity), and anti-β-actin (1:10000, Abconal). After washing, the membranes were incubated with HRP-conjugated secondary antibodies (1:2000) for 1 h at room temperature. Protein bands were visualized using an Enhanced Chemiluminescence (ECL) detection system. The relative expression levels (fold change) of target proteins were quantified by densitometric analysis using β-actin as an internal control.

### Cell Invasion Assay

Cell invasion was evaluated using Transwell chambers pre-coated with Matrigel (Solarbio). Briefly, the upper surface of the Transwell membrane was coated with Matrigel (1:4 dilution) and incubated at 37 °C for 4 h. Cells were harvested and seeded into the upper chamber (2х10^^5^cells/well). The lower chamber was filled with complete medium containing 10% FBS. After 48 h of incubation, the non-invasive cells on the upper surface were gently removed with cotton swabs. The cells that had invaded the lower surface were fixed with 4% paraformaldehyde for 30 min and stained with 0.1% crystal violet for 20 min. The number of invading cells was quantified by counting three random fields under a light microscope (Olympus).

### Cell Cycle Analysis

Cell cycle distribution was determined by flow cytometry after transfection for 48 h. Briefly, cells were harvested by trypsinization and collected by centrifugation. The cell pellets were washed with ice-cold PBS and then fixed in pre-cooled ethanol for 12–24 h, following by resuspending in propidium iodide (PI) staining solution. The samples were incubated at 37 °C for 30 min in the dark and were analyzed using a flow cytometer (Beckman).

#### Measurement of iron, MDA and GSH levels

Cellular supernatant was collected after tranasfection for 48 h to determine the iron (Fe^2+^), MDA and GSH concentrations according to the manufacturer’s instructions for the test kits. The optical density of iron, MDA, and GSH was detected using a microplate reader (Thermo Scientific, China). The concentrations of iron, MDA, and GSH were normalized to the total protein content.

### RNA Extraction and Real-time PCR Assay

Cellular samples were collected for real-time PCR assay. Additionally, tumor tissues and paired control samples were collected from 15 HCC patients admitted to the Department of Hepatobiliary and Pancreatic Surgery, Zhongda Hospital, Southeast University (Nanjing, China) between April and August 2020. All samples were firstly snap-frozen in liquid nitrogen and stored. RNA samples were isolated using RNAiso Plus reagent (Takara, Japan) and reverse-transcribed with a PrimeScript™RT Master Mix kit (Takara). Quantitative PCR was performed on a LongGene Q2000B real-time PCR instrument (LongGene, China). Gene-specific primers (Table 1) were synthesized by Shanghai Sangon Biotechnology Co. Ltd. (China). RNA relative expression levels were calculated using the 2^−△△Ct^ method, with GAPDH as the endogenous control. An ethical approval was obtained prior the collection of samples.

**Table 1:** The sequences of specific primers used in this study

Primers	Specific sequence (5’-3’)
*GAPDH*-hF	TGACAACTTTGGTATCGTGGAAGG
*GAPDH*-hR	AGGCAGGGATGATGTTCTGGAGAG
*CDKN2A*-hF	CTTCCTGGACACGCTGGT
*CDKN2A*-hR	CAAACCCACAAATGGTTTCC
*HELLS*-hF	GCTTGATGGGTCCATGTCTT
*HELLS*-hR	GACATCTATCCTGGGCCTGA
*CDCA7*-hF	CTTGTCATCAATGCCGTCAG
*CDCA7*-hR	CAGTTGCAGATTCCTCGACA
*DDX11-AS1*-hF	ACCTTTGAAACGGACGACAC
*DDX11-AS1*-hR	TAGGGAGGTGAGGTGATTCG

F, forward primer. R, reverse primer. H, human

### Validation of the Protein Levels

To further confirm protein-level dysregulation, immunohistochemistry data for selected proteins were obtained from the Human Protein Atlas [16] for comparative analysis between HCC and normal liver tissues.

### Statistical Analysis

SPSS 22.0 (IBM, USA) was used for statistical analysis. Differences between groups were analyzed using the t-test. Univariate and multivariate Cox regression analysis was employed to evaluate the association of factors with HCC prognosis in the TCGA LIHC patientsm. Hazard ratio (HR) with 95% confidence interval (CI) was reported. Differences between different cell groups were analyzed using the ordinary one-way ANOVA (Tukey’s multiple comparisons test, GraphPad Prism 8.0, GraphPad, USA). The significant criterion was set at *p* < 0.05.

## Results

### Differential Expression Analysis

**Fig. 1:**
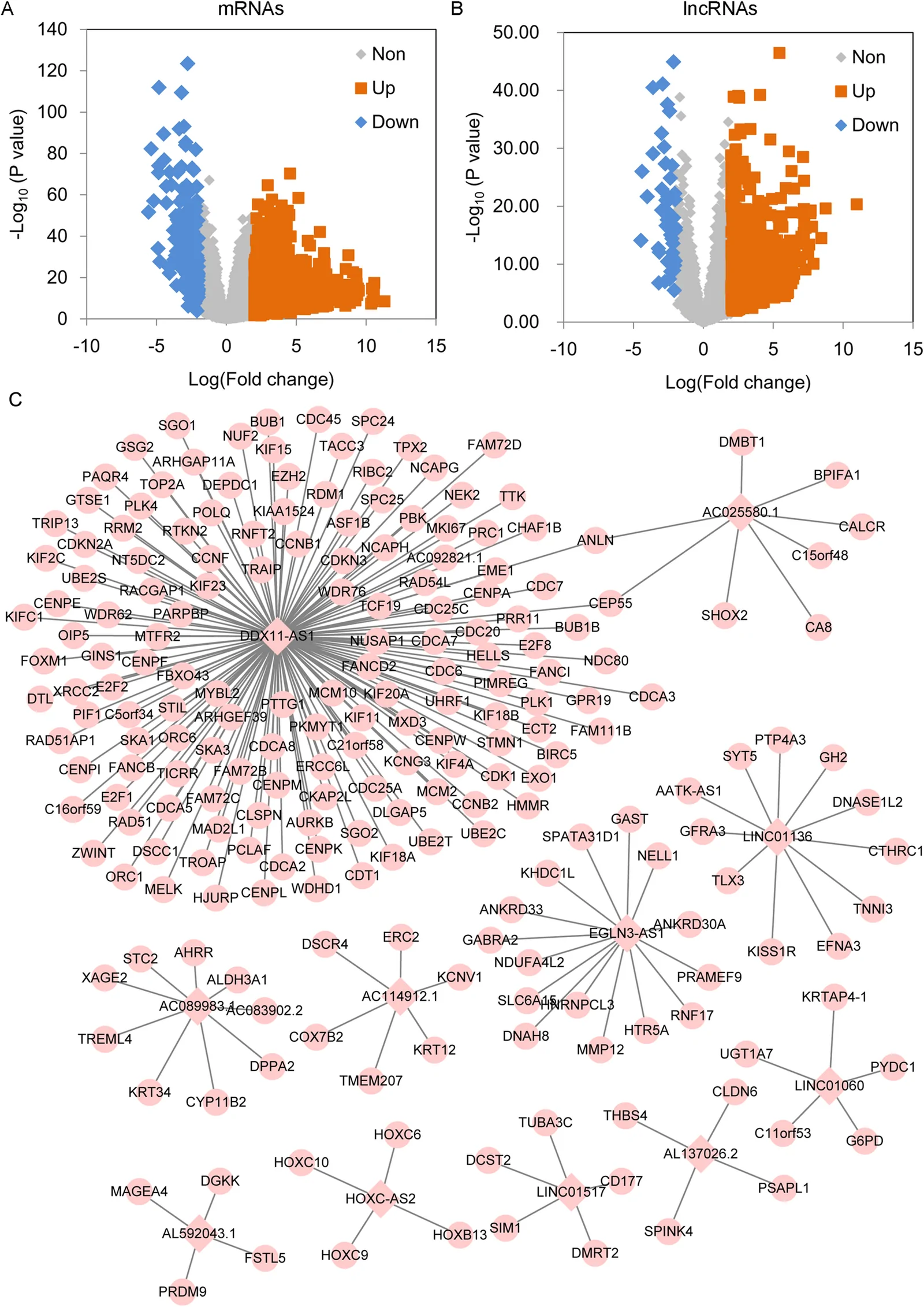
The lncRNA-mRNA regulatory networks of ferroptosis related genes. **A** and **B**, the volcano plots of DEGs and DElncRNAs, respectively. Orange and blue dots represent upregulation and downregulation, respectively. **C**, the lncRNA-mRNA regulatory networks. Diamond indicates lncRNAs, and circles represent mRNAs (genes), respectively. All the nodes were upregulated

We identified 1999 DEGs (1794 up and 205 down) and 1092 DElncRNAs (1034 up and 58 down) in HCC tissues compared to adjacent tissues **(**Fig. 1A and B).

### Identification of the Ferroptosis-related RNAs

Co-expression analysis identified 218 significant lncRNA-mRNA pairs, consisting of 11 DElncRNAs and 216 DEGs (Fig. 1C). Among these, the upregulated lncRNA *DDX11*-*AS1* exhibited the highest connectivity, co-expressed with 147 targets, including *CDK1*, *CDKN2A*, *EZH2*, *CDC25A*, and *FOXM1*.

**Fig. 2:**
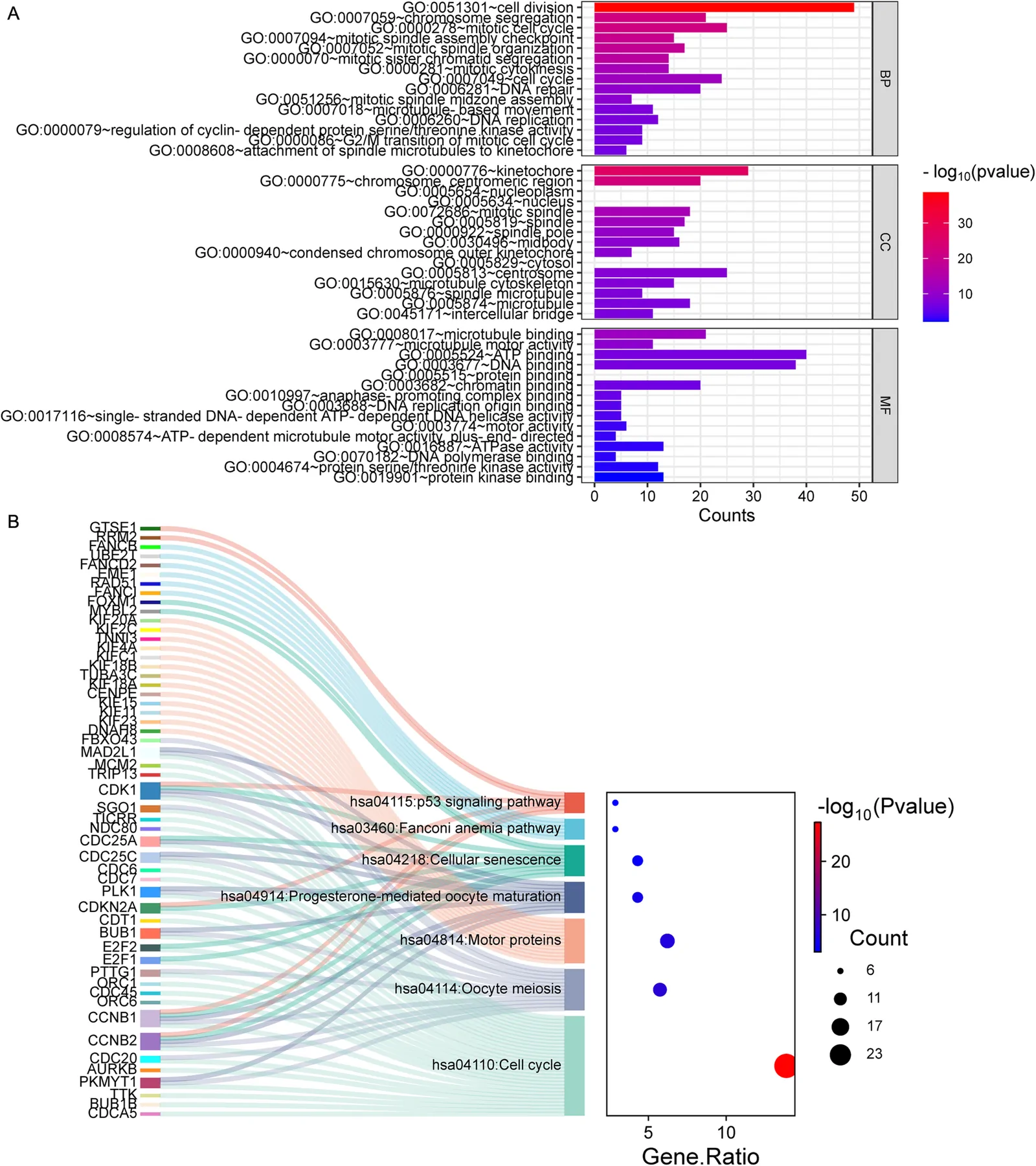
Functional enrichment analysis. **A**, the function terms (top 15) significantly associated with genes. **B**, the Sankey diagram indicates the significant KEGG pathways associated with genes. The bubble diagram (right) indicates the significance (color) and gene number (diameter) enriched in corresponding pathways

Functional enrichment analysis of the 216 DEGs highlighted their involvement in cell division, chromosome segregation, and DNA repair (Fig. 2A). At the cellular component level, these genes were linked to the nucleus and mitotic spindle. Molecular functions included microtubule binding, ATP binding, chromatin binding, and protein binding were associated with these DEGs. KEGG pathway analysis linked these genes with 7 KEGG pathways, including cell cycle, cellular senescence, and the p53 pathway (Fig. 2B).

### Ferroptosis-related RNA Signature in HCC

**Fig. 3:**
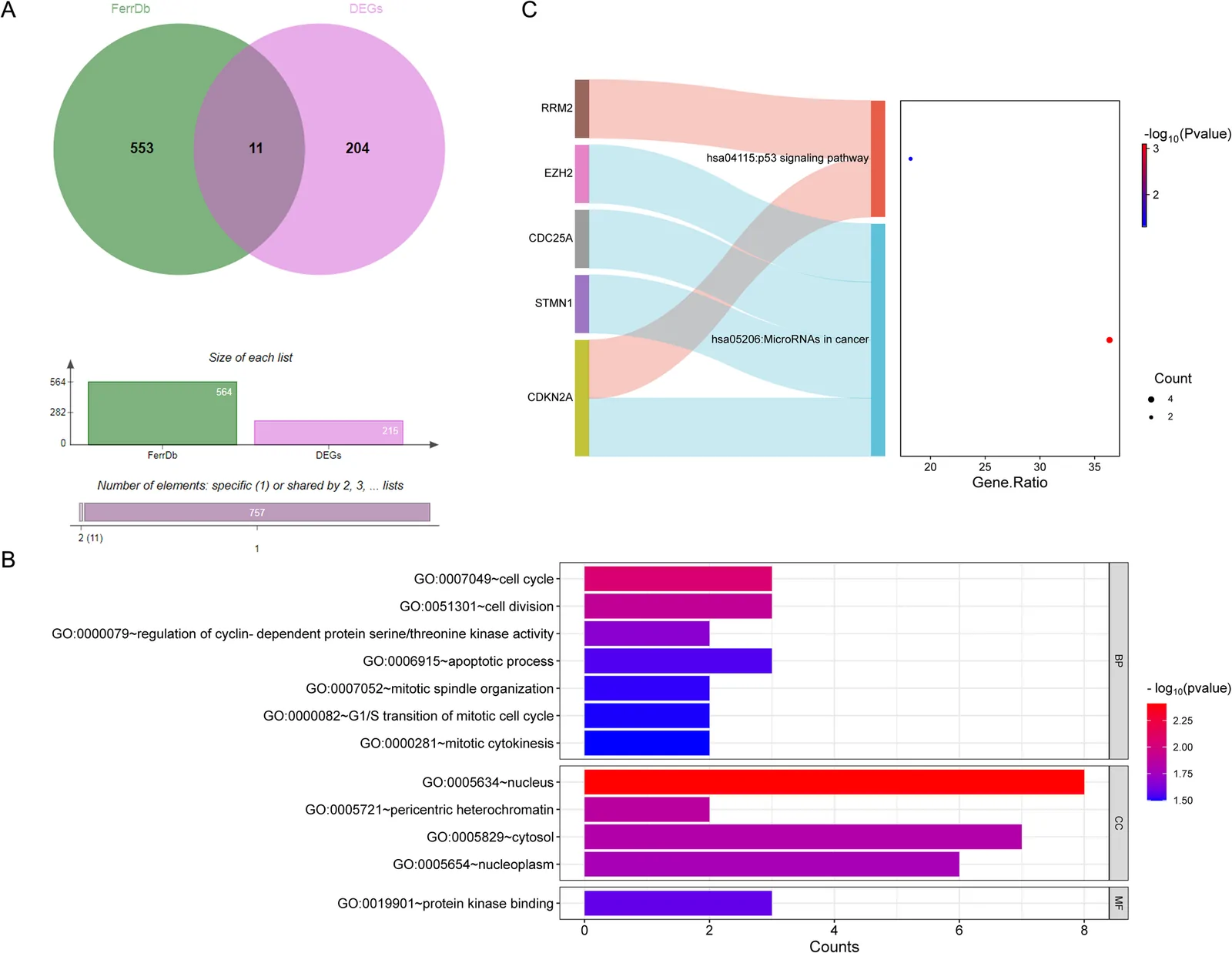
Selection and enrichment analysis of the hub ferroptosis related genes. **A**, the Venn diagram showing the intersection. **B**, the GO terms. C, a Sankey diagram indicates the KEGG pathways significantly associated with the 11 genes

**Fig. 4:**
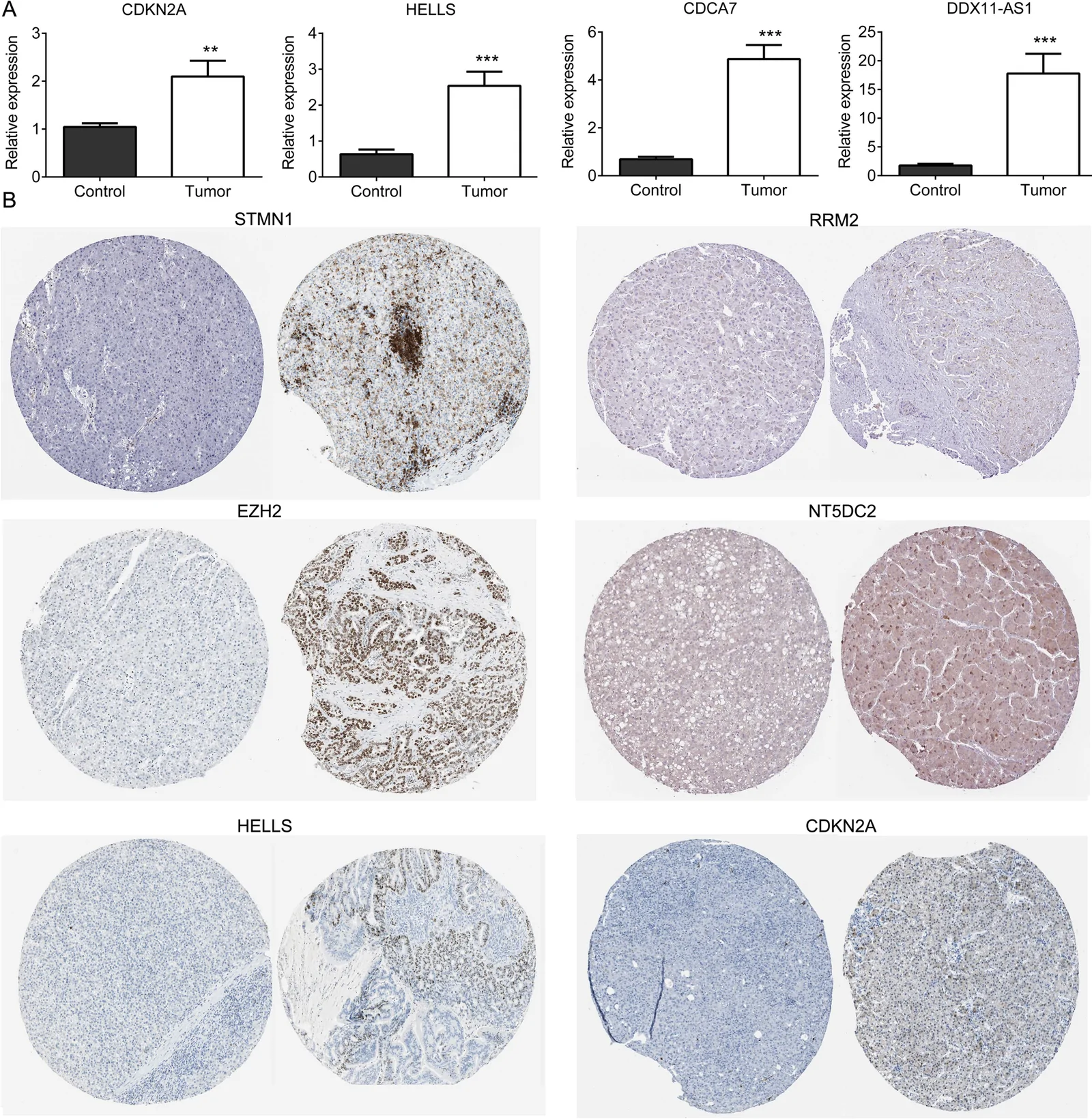
Validation analysis for the hub ferroptosis related genes. **A**, expression levels of genes between HCC tumor samples and normal controls. Differences between groups were analyzed using the t-test. **B**, expression levels of hub proteins between HCC and normal tissues by immunohistochemistry were downloaded from the Human Protein Atlas

We retrieved 564 ferroptosis-related genes from FerrDb, including 264 drivers, 11 markers, 238 suppressors, and 62 unclassified regulators. Among these, 11 genes overlapped with the 216 previously identified DEGs (Fig. 3A**)**, including *CDC25A*, *CDCA7*, *CDKN2A*,* FANCD2*, *HELLS*, *EZH2*, *CDCA3*, *KIF20A*, *NT5DC2*, *RRM2*, and *STMN1*. All 11 ferroptosis-related DEGs exhibited positive co-expression with *DDX11*-*AS1* (Fig. 1B). Functional analysis linked these genes to the GO biological processes of cell division and cell cycle (Fig. 3B) and KEGG pathways of the p53 signaling pathway (Fig. 3C**)**. Experimental validation by RT-qPCR assay confirmed significant upregulation of *CDCA7*, *CDKN2A*, *HELLS*, and *DDX11*-*AS1* in HCC tumor samples (Fig. 4A**)**. Consistent with mRNA findings, protein expression levels of *RRM2*, *STMN1*, *EZH2*, *NT5DC2*, *HELLS*, and *CDKN2A* from the HPA showed elevated levels in tumor samples **(**Fig. 4B).

### Prognostic Value of Ferroptosis-Related RNAs

**Fig. 5:**
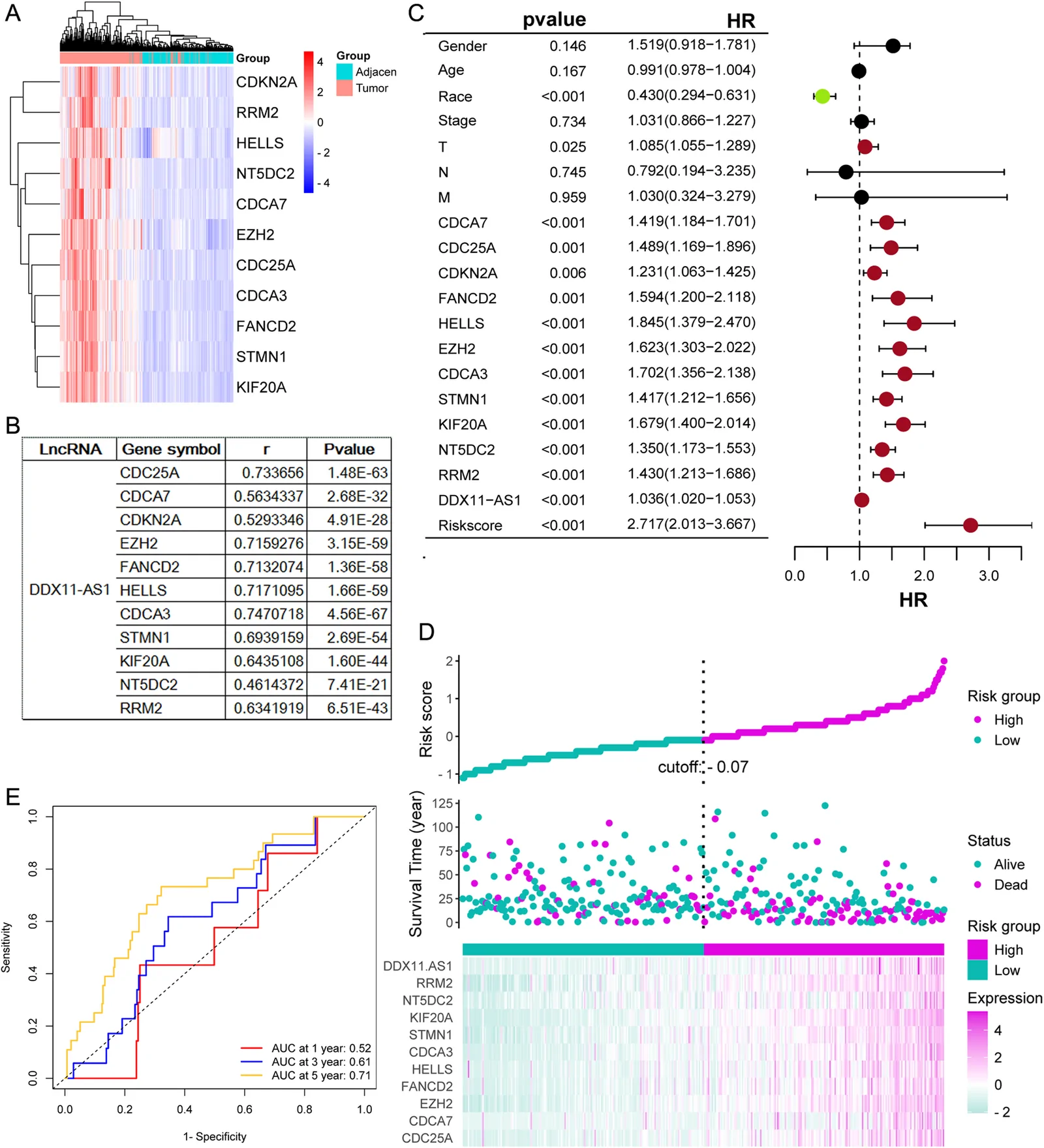
Association of 11 hub genes with the prognosis of HCC. **A**, the clustering of 11 hub ferroptosis-related genes. **B**, COX regression analysis for RNA expression levels in HCC prognosis, and the forest plot (**C**). **D**, distribution of risk score, survival status, and the expression of the 11 hub genes. **E**, ROC curves showing the accuracies of the risk score in predicting the 1, 3, and 5-year survival probabilities

In the independent GSE36376 dataset, all 11 genes were upregulated in tumor samples (Fig. 5A and Supplementary Figure S1), and correlated with *DDX11*-*AS1* (*r* > 0.5 and *p* < 0.05, Fig. 5B). Univariate COX analysis showed that all the 11 genes and the riskscore based on them were associated with HCC prognosis significantly (Fig. 5C). However, multivariate analysis showed none of them, except for riskscore, was an independent risk factor for HCC survival (Supplementary FigureS2). ROC analyssi showed that the riskscore derived from the expression levels of these 11 genes and *DDX11*-*AS1* demonstrated moderate predictive power for overall survival probability (Fig. 5D and E).

### Construction of a Ferroptosis-Associated ceRNA Network

**Fig. 6:**
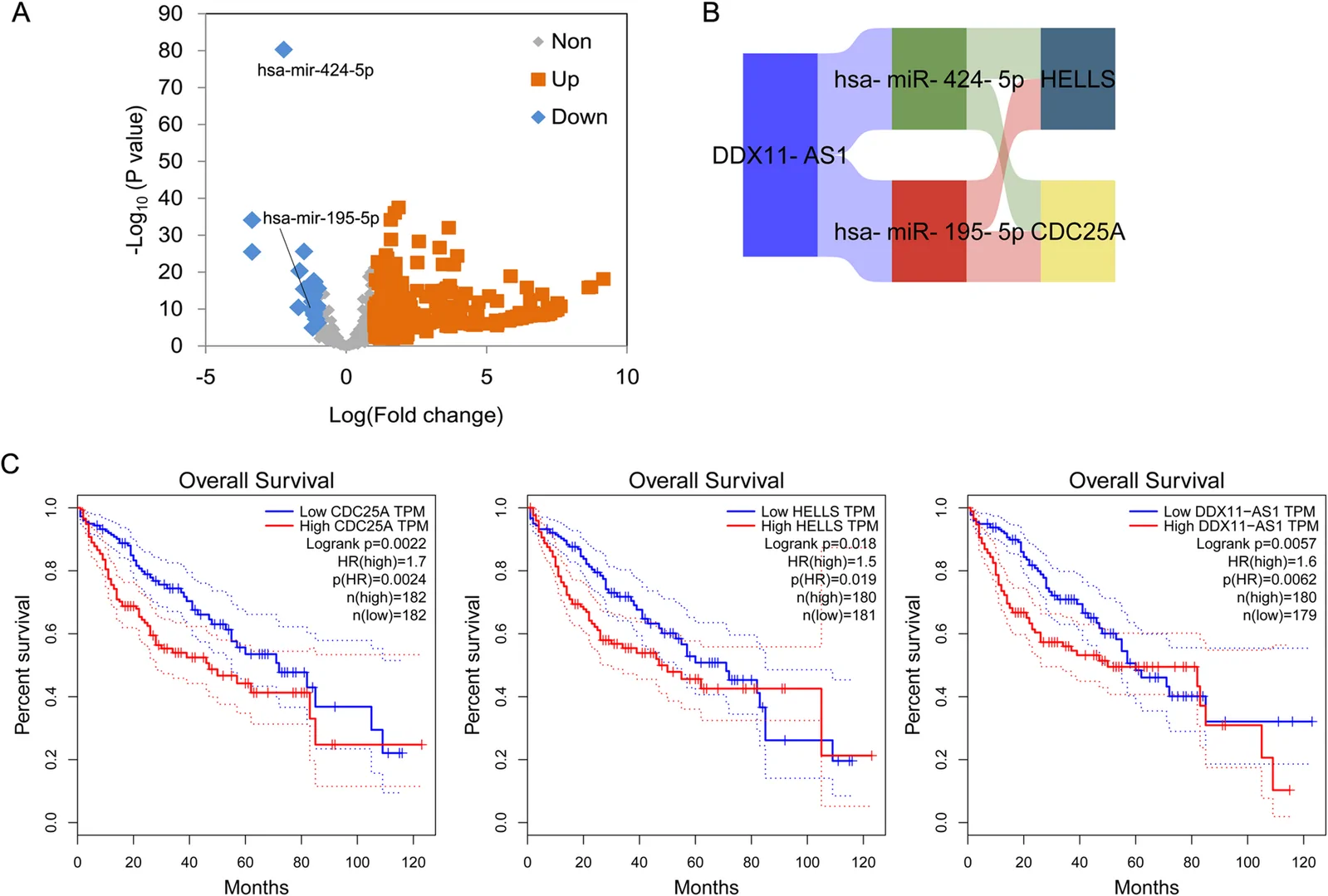
Association of genes with immune infiltrating levels and the ceRNA network. **A**, the heatmap showing the association of the infiltrating levels of immune cells with gene expression levels. **B**, the volcano plot showing the differentially expressed miRNAs (|log2fold change|>1 and adjust. *p* < 0.05) in the TCGA LIHC samples. Orange, blue, and grey dots represent upregulation, downregulation, and non-differentially expression, respectively. **C**, a Sankey diagram showing the ceRNA regulatory network. **C**, overall survival analysis in LIHC patients. The analysis was performed using the GEPIA2.0 online tool. In Kaplan-Meier survival curves, red and blue curves separately represent high-expression group and low-expression group, respectively

To elucidate the post-transcriptional miRNA-mRNA regulation of the 11 ferroptosis-related genes, we identified 22 downregulated miRNAs in the TCGA LIHC cohort (Fig. 6A), two miRNAs (*miR-195* and *miR-424*) were found to correlate with *DDX11*-*AS1* and target DEGs. Finally, a ceRNA network comprises four miRNA-mRNA regulatory pairs involving two miRNAs (*miR-195* and *miR-424*), two DEGs (*CDC25A* and *HELLS*), and one lncRNA *DDX11-AS1* was constructed (Fig. 6B). Notably, both *miR-195* and *miR-424* regulated *CDC25A* and *HELLS*.

### Association of RNA Expression with HCC Patient Survival

Survival analysis performed via GEIPA2 showed that elevated expression of *DDX11*-*AS1*, *CDC25A*, and *HELLS* were significantly related to reduced overall survival in HCC patients (Fig. 6C). A similar prognostic pattern was observed for *CDCA3*, *CDKN2A*, *EZH2*, *FANCD2*, *KIF20A*, *NT5DC2*, *RRM2*, and *STMN1* (Supplementary Figure S3). These results showed the potential prognostic value of this RNA signature in HCC.

### Knockdown of *DDX11*-*AS1*, *CDC25A*, and *HELLS* Promoted Ferroptosis in HCC Cells

**Fig. 7:**
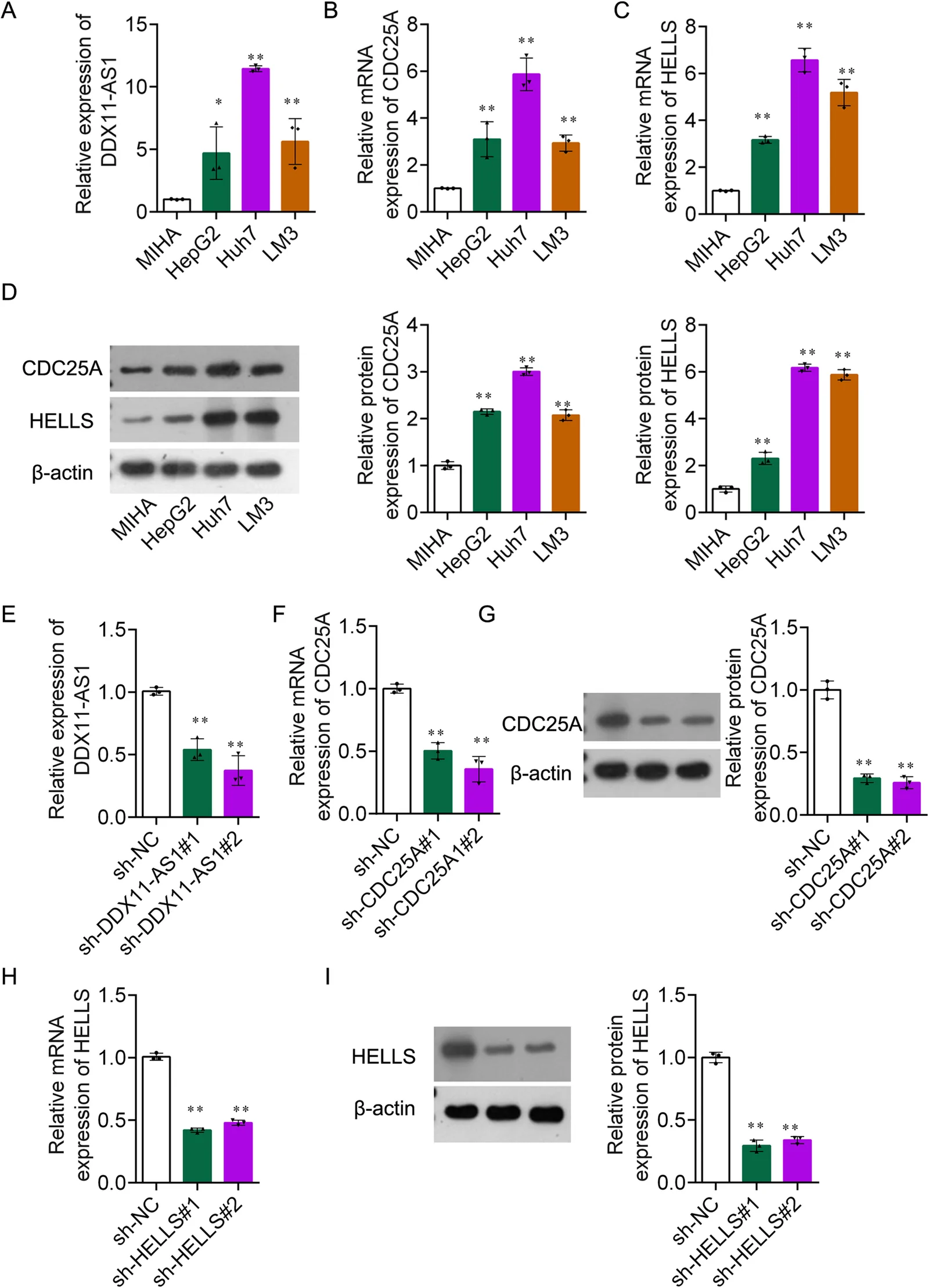
Relative expression levels of mRNAs and proteins of DDX11-AS1, CDC25A, and HELLS. **A**-**C**, the relative expression level of *DDX11-AS1* and mRNA levels of *CDC25A* and* HELLS* genes. **D**, the protein levels of CDC25A and HELLS. **E**-**I**, the relative expression levels of mRNAs and proteins of DDX11-AS1, CDC25A, and HELLS following the knockdown transfections. * and ** indicates *p* < 0.05 and 0.01 vs.control (MIHA or sh-NC), respectively

**Fig. 8:**
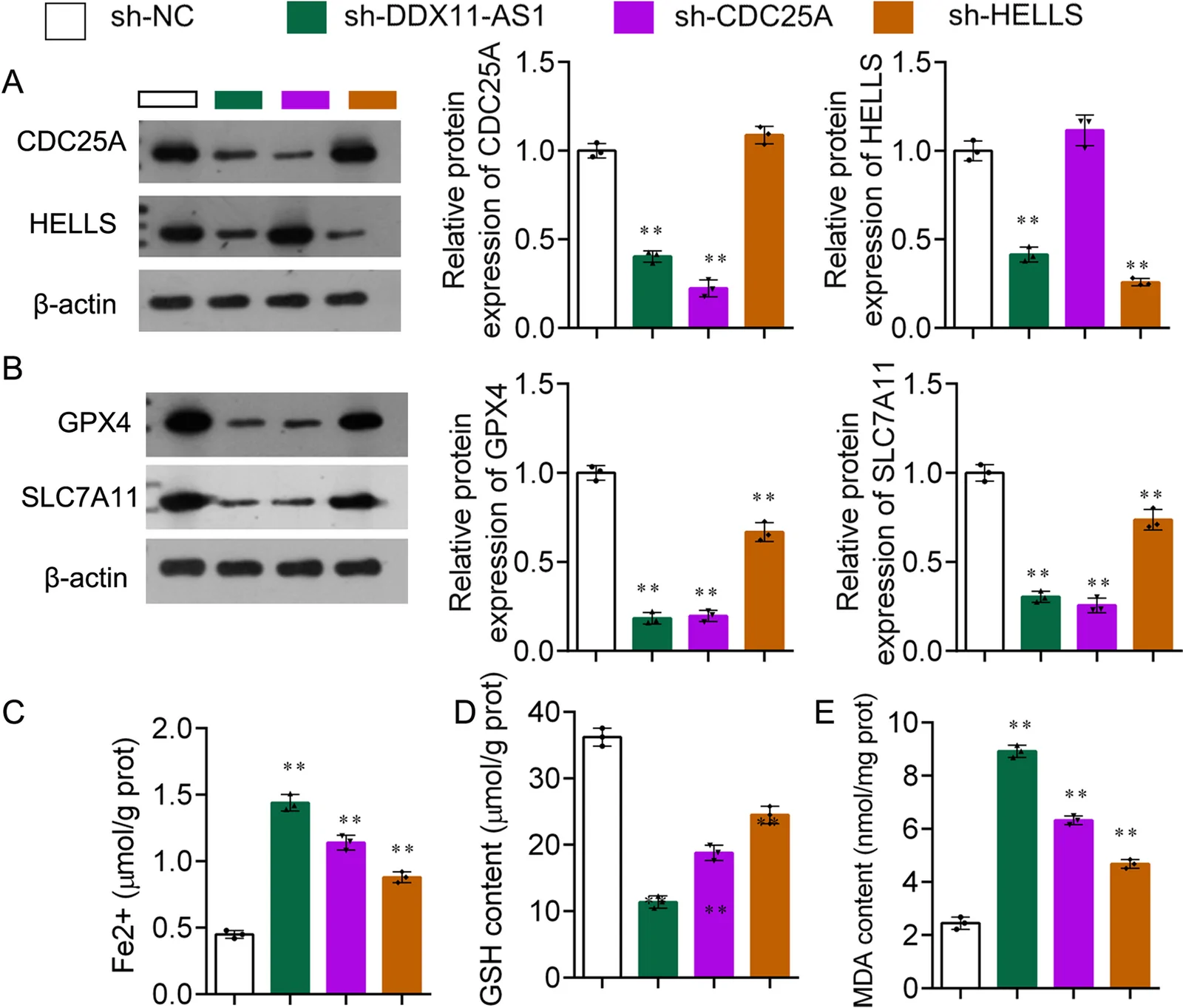
Inhibition of *DDX11-AS1*, *CDC25A*, and *HELLS* promotes HCC cell ferroptosis. **A**, the relative protein expression levels of CDC25A and HELLS following transfections. **B**, the protein levels of GPX4 and SLC7A11 following transfections. **C**-**E**, the Fe^2+^, GSH, and MDA levels in Huh7 cells following the knockdown of *DDX11-AS1*, *CDC25A*, and *HELLS*. ** indicates *p* < 0.01 vs. sh-NC

**Fig. 9:**
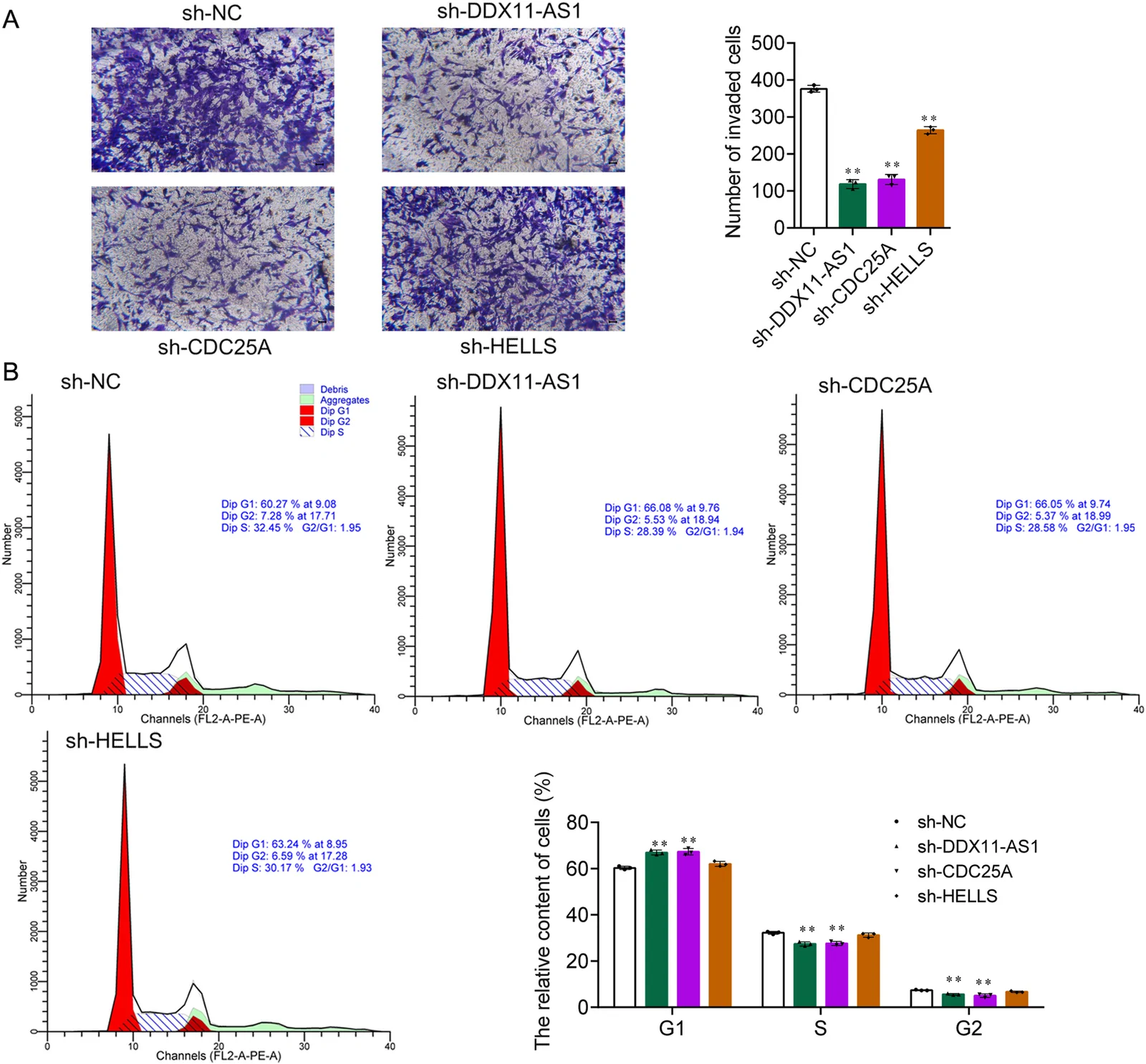
Inhibition of *DDX11-AS1*, *CDC25A*, and *HELLS *suppresses HCC cell invasion and alters cell cycle distribution. **A**, the invasion of Huh7 cells following transfections for 48 h. **B**, the cell cycle distribution ratio of Huh7 cells following transfections for 48 h. ** indicates *p* < 0.01 vs. sh-NC

To validate the functional roles of these ferroptosis-related genes, we inhibited the expression of *DDX11*-*AS1*, *CDC25A*, and *HELLS* (all identified as potential ferroptosis suppressors) in HCC cell lines. Initial validation revealed that these three RNAs were significantly upregulated in HCC cell lines HepG2, Huh7, and LM3 compared to controls (Fig. 7A-D). Subsequently, shRNA-mediated knockdown of *DDX11*-*AS1*, *CDC25A*, and *HELLS* were performed in Huh7 cells (Fig. 7E-I). We observed that transfection with sh-*DDX11-AS1*, sh-*CDC25A*, or sh-*HELLS* suppressed the protein expression of the ferroptosis markers *GPX4 *and *SLC7A11 ***(**Fig. 8A-B**)**, This was accompanied by a decrease in GSH content and a significant increase in Fe^2 +^ and MDA levels in Huh7 cells **(**Fig. 8C-E). Furthermore, the knockdown of these targets resulted in a marked reduction in the number of invaded cells (Fig. 9A). To investigate the impact on cell cycle progression, we analyzed the cell cycle distribution following knockdown of sh-*DDX11*-*AS1*, sh-*CDC25A*, and sh-*HELLS*. The results showed that silencing *DDX11-AS1* or *CDC25A* increased the proportion of cells in the G1 phase while decreasing the populations in the S and G2 phases (Fig. 9B). Collectively, these results showed that sh-*DDX11*-*AS1*, sh-*CDC25A*, and sh-*HELLS* inhibited cell proliferation and invasion by promoting ferroptosis in Huh7 cells.

## Discussion

This study identified 1999 DEGs and 1092 DElncRNAs, including 11 ferroptosis-related genes. Univariate Cox regression analysis showed that 11 ferroptosis-related genes (*CDC25A*, *CDCA7*, *CDKN2A*,* FANCD2*, *HELLS*, *EZH2*, *CDCA3*, *KIF20A*, *NT5DC2*, *RRM2*, and *STMN1*) and the co-expressed lncRNA *DDX11*-*AS1* was associated with HCC prognosis. Elevated expression levels of *CDC25A*,* CDKN2A*,* FANCD2*,* EZH2*,* HELLS*, and *DDX11*-*AS1* might contribute to poorer over survival. Finally, in vitro cell validation experiments showed that sh-*DDX11*-*AS1*, sh-*CDC25A*, and sh-*HELLS* inhibited cell proliferation and invasion via promoting ferroptosis in Huh7 cells, suggesting the functional regulation of these genes in HCC might by regulating ferroptosis.

Among those ferroptosis-related genes, *CDC25A* and *HELLS* appear to be post‑transcriptionally regulated by *DDX11-AS1* through competitive binding to *miR-195* and *miR-424*. Previous studies had reported the association of both miRNAs with the prognosis, invasion, clinical stage, and recurrence of human cancers, including HCC [17]. *HELLS* is known to control mitochondrial dynamics, chromatin stability in liver cancer [18], and its knockdown increased histone 3 lysine 9 trimethylation (H3K9me3) in liver cancer cells [18]. *DDX11-AS1* acts as a novel oncogenic lncRNA that promotes HCC progression via miRNAs such as *miR-195-5p* and *miR-873-5p* [19, 20] or through direct interaction with genes like *EZH2* [6, 21]. *DDX11-AS1* expression correlates with HCC progression and poor prognosis and therefore is a potential therapeutic target [22]. Notably, this lncRNA has been reported to repress sorafenib-induced ferroptosis in HCC by stabilizing Nrf2 and promoting its nuclear translocation [23]. Our present study extends these observations by demonstrating that *DDX11*-*AS1* may influence HCC prognosis through a ceRNA network involving ferroptosis-related genes *CDC25A* and *HELLS* via sponging *miR-195/424*. In vitro cellular experiments in Huh7 cells showed that *CDC25A* and *HELLS* knockdown suppressed cell invasion and the ratios of cell distributed at the S and G2 phases, and promoted ferroptosis. These findings collectively highlighted the significance of these ferroptosis-associated RNAs in HCC pathogenesis and progression.

*EZH2* is consistently upregulated in HCC and functions as a core component and interplays with PRC2 to epigenetically regulate histone methyltransferase activity by promoting H3K27me3 [10, 24]. *EZH2* promotes the progression of HCC by epigenetic regulation of targets non-coding RNAs [25–27]. Among the targets of EZH2, ferroptosis-related genes occupy a significant proportion. Ferroptosis interacts with the tumor immune microenvironment and promotes tumor progression [7, 8]. EZH2/H3K27me3 repressed *SLC7A11* and the later modulated ferroptosis in various diseases [28, 29]. Recent studies further highlighted the role of other identified DEG) in ferroptosis. *CDKN2A* has emerged as a novel regulator of ferroptosis, potentially through its influence on lipid metabolism [30]. In glioblastoma, deletion of *CDKN2A* reduces the formation of lipid droplets, which makes it susceptible to lipid peroxidation and ferroptosis [30]. Also, the regulation of *CDC25A* on ferroptosis is context‑dependent: it decreased Fe^2 +^ levels, increased glutathione, superoxide dismutase, and promoted ferroptosis in HUVECs [31], its overexpression inhibited ferroptosis in OSAHS-hypertension model [31]. These findings showed ferroptosis as a potential therapeutic target in HCC, with DEGs including *CDC25A* and *EZH2* acting as key regulatory factors. Based on the above analysis, we speculated that these ferroptosis-related genes may reveal novel mechanistic and therapeutic insights in HCC progression.

Based on the functional enrichment analysis, we found that the ferroptosis-related DEGs were associated with the biological processes or pathways related to cell division and cell cycle. The associations of DEGs *CDC25A*, *CDCA7*, *CDKN2A*, *FANCD2*, *HELLS*, and *EZH2* with cell cycle have been widely reported [32, 33]. *FANCD2* regulates the cell cycle through CDK-mediated phosphorylation at the S592 site [32]. *FANCD2* depletion significantly suppresses cell proliferation, cell cycle progression, tumor colony formation and metastasis [32]. Downregulation of *CDCA7* and *CDC25A* suppresses cell proliferation and cell cycle of cancer cells [34–36]. The interaction of them with ferroptosis might show that ferroptosis plays important roles in regulating cell cycle during HCC progression.

Recent evidence shows that ferroptosis associates with immune infiltration tightly [37–39]. *FANCD2*, a ferroptosis regulator, was reported to be related to a higher tumor immune dysfunction and exclusion score in lung adenocarcinoma and HCC [37, 39]. Tang et al. showed that elevated *FANCD2* level linked to a higher TIDE score in HCC patients [39]. Xu et al. identified a immune prognostic model related to *EZH2* in clear cell renal cell carcinoma [40]. Also, it predicted a poor prognosis and high level of T-cell infiltration in oral squamous cell carcinoma [41]. The correlation of genes with immune cell infiltration in HCC might provide novel insights into the immunotherapy for this disease.

However, there are some limitations in this study. The primary implication of this limitation is that our findings remain hypothetical and correlative, lacking in vivo validation in animal models for the functions of these ferroptosis-related DEGs, including *CDC25A* and *HELLS*. However, the in vitro cellular experiments using HCC cells lines showing that sh-*DDX11*-*AS1*, sh-*CDC25A*, and sh-*HELLS* inhibited cell proliferation and invasion, and promoted Fe^2 +^ accumulation in Huh7 cells Actions, including functional validation in animal models, are urgently recommended to transform this prediction into actionable knowledge.

## Conclusions

In conclusion, this study identified 11 ferroptosis-related DEGs that play crucial roles in the pathogenesis and prognosis of HCC. Through ceRNA network analysis we demonstrated that genes including *CDC25A*, *CDCA7*, *CDKN2A*, *FANCD2*, *RRM2*, *STMN1*, *HELLS*, and *EZH2* genes were potentially regulated by the oncogenic lncRNA *DDX11*-*AS1* via sponging *miR*-*195* and *miR*-*424*. These DEGs were associated with cell cycle or cell division to participate in the progression of HCC. Elevated expression levels of these RNAs were associated with poorer outcomes, further supporting their contributory role in tumor aggressiveness. In vitro cellular experiments in Huh7 cells showed that knockdown of *DDX11*-*AS1*, *CDC25A*, and *HELLS* inhibited cell proliferation and invasion via promoting ferroptosis in Huh7 cells. Collectively, our findings provide novel mechanistic insights into HCC, adding the importance of ferroptosis-associated regulatory networks in tumor development and patient prognosis.

## Supplementary Information

Below is the link to the electronic supplementary material.

Supplementary Material 1

Supplementary Material 2

Supplementary Material 3

## Data Availability

The data generated or analyzed during the current study are available from the corresponding author on reasonable request. The original TCGA dataset is available at The Cancer Genome Atlas (TCGA, [https://cancergenome.nih.gov/](https:/cancergenome.nih.gov) ). The microarray dataset GSE36376 (GPL10558) was obtained from the Gene Expression Omnibus (https://www.ncbi.nlm.nih.gov/geo/).
